# Postexercise muscle oxygen uptake kinetics in older breast cancer survivors and healthy individuals: Association with myosteatosis

**DOI:** 10.1113/EP093370

**Published:** 2026-01-08

**Authors:** Nathan R. Weeldreyer, Thomas J. McMurtry, Stephen J. Foulkes, Amanda Shewman, Edith Pituskin, Rachel J. Skow, Richard B. Thompson, Justin Grenier, Deirdre E. O'Neill, Robert C. Welsh, Mark J. Haykowsky, Corey R. Tomczak

**Affiliations:** ^1^ Integrated Cardiovascular Exercise Physiology (iCARE) Laboratory, College of Health Sciences, Faculty of Nursing University of Alberta Edmonton Alberta Canada; ^2^ Heart, Exercise and Research Trials Lab St Vincent's Institute of Medical Research Fitzroy Victoria Australia; ^3^ Department of Radiology and Diagnostic Imaging, Faculty of Medicine & Dentistry, College of Health Sciences University of Alberta Edmonton Alberta Canada; ^4^ Faculty of Medicine & Dentistry, College of Health Sciences University of Alberta Edmonton Alberta Canada; ^5^ Hochgebirgsklinik, Medicine Campus Davos Davos Switzerland; ^6^ College of Kinesiology University of Saskatchewan Saskatoon Saskatchewan Canada

**Keywords:** breast cancer, muscle composition, plantar‐flexion exercise

## Abstract

Reduced cardiorespiratory fitness is common among breast cancer survivors and, although traditionally attributed to cardiac dysfunction, might also be related to peripheral skeletal muscle abnormalities. We examined peak and submaximal plantar‐flexion exercise and recovery kinetics for lower‐leg oxygen uptake (${\dot{V}}_{O_{2}}$), blood flow and arteriovenous O_2_ difference in 35 older, female long‐term breast cancer survivors (age, 70 ± 5 years; 14 ± 6 years post‐treatment) and 19 age‐ and sex‐matched healthy control subjects using MRI. The calf intermuscular fat to skeletal muscle ratio was evaluated using fat‐ and water‐separated MRI to quantify myosteatosis. No significant differences were found between groups for lower‐leg ${\dot{V}}_{O_{2}}$, blood flow or arteriovenous O_2_ difference at peak or during submaximal plantar‐flexion exercise. Recovery kinetics for these measures were similar between groups (all *P *> 0.05). No differences were found in calf muscle mass between breast cancer survivors and control subjects. A higher intermuscular fat to skeletal muscle ratio was significantly associated with reduced peak (cycle exercise) pulmonary ${\dot{V}}_{O_{2}}$ (*r* = −0.37, *P* = 0.01), lower plantar‐flexion power output (*r* = −0.325, *P* = 0.012) and delayed muscle ${\dot{V}}_{O_{2}}$ recovery kinetics in both groups (*r* = −0.325, *P* = 0.015). Older, female long‐term breast cancer survivors have comparable lower‐limb skeletal muscle oxidative capacity to control subjects. However, myosteatosis represents a key determinant of exercise performance and recovery kinetics regardless of breast cancer history. Interventions targeting skeletal muscle quality might be effective in improving both submaximal and maximal exercise tolerance and recovery kinetics in older women with or without a breast cancer diagnosis.

## Abstract (highlights)

- **What is the central question of this study?**
  Do older breast cancer survivors have impaired lower‐leg muscle peak oxygen uptake and recovery kinetics compared with control subjects?
- **What is the main finding and its importance?**
  Older breast cancer survivors showed no significant differences from control subjects in lower‐leg oxygen uptake or recovery kinetics following peak and submaximal plantar‐flexion exercise. Greater calf myosteatosis was significantly linked to lower peak (cycle exercise) pulmonary oxygen uptake, reduced lower‐leg (plantar‐flexion exercise) oxygen uptake and slower recovery kinetics in both groups. These findings highlight the impact of muscle composition (particularly myosteatosis) on exercise capacity and recovery in older women with or without a breast cancer diagnosis.

## 1 INTRODUCTION

Reduced cardiorespiratory fitness and exertional fatigue are common symptoms among women diagnosed with breast cancer across the survivorship continuum (Bower et al., 2006; Haykowsky et al., 2022; Jones et al., 2012). The lower peak oxygen uptake (${\dot{V}}_{O_{2}}$) has traditionally been attributed to cardiac dysfunction resulting from the adverse cardiotoxic effects of cancer therapy, underlying cardiovascular disease and sedentary deconditioning (Foulkes, Haykowsky et al., 2024; Haykowsky et al., 2022; Kirkham et al., 2019). However, emerging evidence suggests that skeletal muscle abnormalities might contribute to reduced peak ${\dot{V}}_{O_{2}}$ in breast cancer survivors (BCS) (Beaudry et al., 2023, 2020; Foulkes, Haykowsky et al., 2024; Haykowsky et al., 2022; Mijwel et al., 2018).

Studies examining exercise limitations in BCS have focused primarily on maximal whole‐body exercise (Haykowsky et al., 2022; Jones et al., 2012). Although this approach has provided valuable insights, it might overlook the peripheral factors that contribute to impaired exercise tolerance. Small muscle mass exercise is a unique model to isolate peripheral limitations by minimizing the influence of central cardiovascular constraints on exercise performance (Foulkes, Wagner et al., 2024; Jendzjowsky et al., 2007). Moreover, because daily activities, such as walking and climbing stairs, consist of short bursts of submaximal effort followed by brief recovery periods, it is important to understand recovery patterns in skeletal muscle oxygenation following exercise. As such, the kinetics of muscle ${\dot{V}}_{O_{2}}$ recovery after exercise can provide important insights into skeletal muscle function and oxidative capacity. In patients with heart failure (a syndrome that older BCS are at elevated risk of developing; Haykowsky et al., 2022), delayed pulmonary ${\dot{V}}_{O_{2}}$ and skeletal muscle ${\dot{V}}_{O_{2}}$ recovery kinetics are associated with impaired exercise tolerance, greater disease severity and reduced survival (Bailey et al., 2018; Belardinelli et al., 1997; Fortin et al., 2015; Thompson et al., 2016). This highlights the importance of investigating the postexercise recovery phase of skeletal muscle ${\dot{V}}_{O_{2}}$ and its Fick determinants (muscle blood flow and arterial to mixed venous O_2_ difference) in BCS, which represents a largely unexplored knowledge gap.

Another limitation of prior acute mechanistic studies was the primary focus on middle‐aged BCS during or in the early period (≤5 years) following anti‐cancer treatment (Foulkes, Haykowsky et al., 2024; Haykowsky et al., 2022; Jones et al., 2012). This is important, because the incidence of functional limitations and cardiovascular disease is most pronounced in long‐term (>5 years) and/or older (>65 years) BCS (Kirkham et al., 2019). Moreover, addressing cardiac limitations without also addressing impairments in the periphery results in markedly attenuated improvements in exercise capacity. In heart failure with preserved ejection fraction, improvements seen in the periphery lead to a 27% increase in exercise capacity, compared with 7% for cardiac improvements (Houstis et al., 2018).

In addition, in the short‐term (∼1 year) following chemotherapy, BCS have increased skeletal muscle fat infiltration (myosteatosis) that is associated with reduced whole‐body peak ${\dot{V}}_{O_{2}}$, lower muscle ${\dot{V}}_{O_{2}}$, impaired oxygen extraction and decreased blood flow (Beaudry et al., 2020). A consequence of these impairments is prolonged ${\dot{V}}_{O_{2}}$ recovery kinetics and diminished capacity for repeated daily activities, such as walking or climbing stairs. Currently, it is unknown whether this relationship exists in older long‐term BCS; therefore, the peripheral mechanisms underpinning the reduced exercise tolerance in older, long‐term BCS survivors remains an important question for reducing breast cancer‐related morbidity.

The aim of this study was to compare muscle ${\dot{V}}_{O_{2}}$ and its Fick determinants during and following maximal and submaximal small muscle mass (plantar‐flexion) exercise between older female BCS and age‐ and sex‐matched control subjects (CON). We hypothesize that: (1) BCS will exhibit reduced lower‐leg ${\dot{V}}_{O_{2}}$ during peak plantar‐flexion exercise and delayed postexercise ${\dot{V}}_{O_{2}}$ kinetics compared with CON, driven by a reduced fractional O_2_ extraction; and (2) higher levels of myosteatosis will be associated with reduced and delayed lower‐leg peak ${\dot{V}}_{O_{2}}$ and recovery kinetics, respectively, in both groups.

## 2 MATERIALS AND METHODS

### 2.1 Ethical approval

This study was conducted at the University of Alberta and received ethical approval from the Health Research Ethics Board of Alberta (HREBA‐CC‐22‐0254). This study conformed to the standards set by the *Declaration of Helsinki*, except for registration in a database. All participants provided written informed consent prior to commencement of any study procedures.

### 2.2 Study participants and recruitment

The inclusion criteria for BCS were as follows: (1) age ≥60 years; (2) diagnosed with early‐stage (I–III) breast cancer; and (3) ≥1 year post‐completion of cardiotoxic treatment, which included anthracycline‐based chemotherapy or trastuzumab‐based biological therapy. Age‐ and sex‐matched CON participants were recruited from the local Edmonton area.

For both groups, the exclusion criteria were as follows: (1) a history of coronary artery disease, heart failure, persistent or permanent arrhythmia (e.g. atrial fibrillation), stroke or chronic obstructive pulmonary disease; (2) signs of myocardial ischaemia during a cardiopulmonary exercise test; (3) contraindications for MRI; (4) musculoskeletal limitations that would prevent exercise testing; and (5) a history of any other cancer diagnosis that required chemotherapy or biological therapy.

### 2.3 Cycle ergometry exercise test

Peak pulmonary ${\dot{V}}_{O_{2}}$ (Vmax Encore229, Carefusion, San Diego, CA, USA) was determined using an incremental ramp protocol via stationary cycle ergometry (Ergoselect II 1200, Ergoline, Blitz, Germany). The exercise protocol entailed 3 min exercise at 20 W followed by 10–15 W/min increases until volitional fatigue or an inability to maintain a pedalling frequency of 60 revolutions/min. The ramp rate was individualized based on the participant's age, body size and reported weekly exercise minutes in order to ensure that participants reached volitional exhaustion within 8–12 min of commencing the incremental protocol. Peak pulmonary ${\dot{V}}_{O_{2}}$ was determined as the highest 30 s average in the last 1 min of exercise.

### 2.4 Physical activity levels

As part of a standardized interview with a member of the research team, participants were asked about their frequency, mode and duration of moderate‐ to vigorous‐intensity exercise to quantify the number of minutes of moderate‐ to vigorous‐intensity physical activity performed per week.

### 2.5 MRI

MRI studies were completed using a 2.89 T Siemens Prisma scanner (Siemens Healthineers, Erlangen, Germany).

### 2.6 Calf muscle composition

Calf muscle composition was evaluated using a multi‐slice, multi‐echo MRI protocol that enabled reconstruction of water‐ and fat‐separated images covering the entire calf muscle via the chemical shift‐encoded method. Image analysis was performed from the first slice showing clear anatomical separation of the tibia and fibula through to the distal end of the gastrocnemius muscle. Skeletal muscle volume and composition were quantified using a semi‐automated approach with custom MATLAB code developed by our group (Beaudry et al., 2020).

### 2.7 Incremental plantar‐flexion exercise test

Participants were positioned supine in the scanner, with their dominant leg secured to an MRI‐compatible plantar‐flexion ergometer (Trispect Module, Ergospect). An incremental‐to‐maximal plantar‐flexion exercise test was undertaken, with the initial power output set at 4 W, increasing by 2 W/min until volitional exhaustion. Immediately at exercise termination, time‐resolved transverse images of the popliteal vein (<1 cm proximal to the patella) were acquired continuously for 230 s (100 images, each acquired in 2.3 s) to measure both lower‐leg blood flow and venous O_2_ saturation, as previously described, using our validated technique (Beaudry et al., 2020; Skow et al., 2025). Briefly, MR susceptometry‐based oximetry was used to measure venous O_2_ saturation based on the magnetic field shift associated with the concentration of deoxyhaemoglobin (Haacke et al., 1997; Mathewson et al., 2015). The same MRI acquisition included simultaneous quantification of blood flow in the popliteal vein (<1 cm proximal to the patella) using phase‐contrast imaging. Images were acquired perpendicular to the popliteal vein (axial image orientation) proximal to the knee.

### 2.8 Submaximal plantar‐flexion exercise

Following a 15 min rest period, participants performed submaximal plantar‐flexion exercise with their non‐dominant limb. The power output was set at 60% of the peak power determined from the incremental test. Time‐resolved transverse images of the popliteal vein were acquired continuously for 230 s during the recovery phase. Data were recorded every 2.3 s, and these data were used for determining the time course of recovery, as noted below.

### 2.9 Calculations

Lower‐leg ${\dot{V}}_{O_{2}}$ was calculated using the Fick principle, incorporating arterial O_2_ saturation from finger pulse oximetry, haemoglobin concentration from a venous blood sample, and lower‐leg blood flow and venous O_2_ saturation derived from MRI, as described above (Beaudry et al., 2020; Skow et al., 2025).

### 2.10 Recovery kinetics of lower‐leg ${\dot{V}}_{O_{2}}$ and its determinants

Postexercise lower‐leg ${\dot{V}}_{O_{2}}$, blood flow and arteriovenous O_2_ difference kinetics were determined starting from the cessation of exercise for a 230 s passive recovery period. Although most participants demonstrated an exponential decline in measured parameters, some demonstrated physiological profiles that were not amenable to monoexponential curve fitting. As such, the half‐time for recovery was determined as we have previously done (Jendzjowsky et al., 2007) using commercially available software (Origin, OriginLab Corporation, Northampton, MA, USA). Specifically, half‐times were calculated as the time to reach 50% of the difference between peak and end recovery values (Jendzjowsky et al., 2007).

### 2.11 Statistical analysis

Statistical analyses were performed using SPSS Statistics, v.30.0 (IBM, Armonk, NY, USA). Data are presented as the mean ± SD unless otherwise specified. The Shapiro–Wilks test was used to assess the normality of the distribution for each outcome variable. Student's independent samples two‐tailed *t*‐tests were used to assess between‐group differences for normally distributed data, whereas the Mann–Whitney *U*‐test was used for non‐normally distributed variables. Fisher's exact tests were used to assess differences in medical history between groups. Pearson or Spearman correlation coefficients were determined to assess the relationships between key physiological variables. The α‐level was set a priori at *P* < 0.05.

## 3 RESULTS

### 3.1 Participant demographics and characteristics

Thirty‐five BCS (mean age, 70 ± 5 years) and 19 CON (mean age, 69 ± 5 years) participated in this study. No significant differences were found between groups for age, body mass (and body mass index), calf muscle mass or myosteatosis. Self‐reported weekly exercise minutes and peak power output or pulmonary ${\dot{V}}_{O_{2}}$ during cycle ergometry exercise testing were also not significantly different between the groups (Table 1).

**TABLE 1:** Participant demographics and characteristics.

Parameter	BCS (*n* = 35)	CON (*n* = 19)	*P*‐value
Age, years	70 ± 5	69 ± 5	0.59
Weight, kg	71.8 ± 14.2	68.6 ± 14.4	0.43
Body mass index, kg/m^2^	27.4 ± 5.6	25.5 ± 5.3	0.24
Calf muscle mass, kg	0.82 ± 0.17	0.76 ± 0.16	0.87
Calf intermuscular fat/skeletal muscle ratio, %	11.8 ± 2.5	11.0 ± 3.0	0.32
Pulmonary ${\dot{V}}_{O_{2}}$ peak, mL/kg/min	19.3 ± 4.6	21.5 ± 5.9	0.28
Peak upright power, W	105 ± 25	116 ± 34	0.23
Self‐reported exercise, min/week	190 ± 189	268 ± 122	0.12
Medical history, *n* (%)			
Hypertension	14 (40)	4 (21)	0.35
Hypercholesterolaemia	10 (29)	3 (16)	0.51
Diabetes	3 (9)	1 (5)	1.00
Medications			
ACE‐i	7	0	–
ARB	6	3	–
β‐Blocker	0	0	–
Calcium channel blocker	2	1	–
Statin	10	2	–
Hypoglycaemic agents	2	0	–
Breast cancer history			
Time post‐treatment, years	14 ± 6	–	–
Cancer staging, *n* (%)		–	–
Stage I	7 (20)	–	–
Stage II	21 (60)	–	–
Stage III	7 (20)	–	–
Receptor status, *n* (%)			
ER and/or PR^+^	23 (66)	–	–
HER2^+^, ER and PR^−^	3 (9)	–	–
HER2 and ER and/or PR^+^	7 (20)	–	–
Triple negative	2 (6)	–	–
Treatment regimen			
Anthracycline chemotherapy, *n* (%)	29 (83)	–	–
Anthracycline dose,^a^ mg/m^2^	255 ± 66	–	–
Anti‐HER2, *n* (%)	8 (23)	–	–
Taxane chemotherapy, *n* (%)	28 (80)	–	–
Platinum‐based chemotherapy, *n* (%)	5 (14)	–	–
Aromatase inhibitors, *n* (%)	14 (40)	–	–
Radiation, *n* (%)	29 (83)	–	–

*Note*: Abbreviations: BCS, breast cancer survivor; CON, control subject; ACE‐i, angiotensin‐converting enzyme inhibitor; ARB, angiotensin receptor blocker; ER, estrogen receptor; HER2, human epidermal growth factor receptor 2; PR, progesterone receptor; ${\dot{V}}_{O_{2}}$, oxygen uptake.

^a^ Doxorubicin equivalent dose.

For the BCS group, the average time since their last adjuvant treatment was 14 ± 6 years. The most frequent receptor status was oestrogen and/or progesterone receptor positivity, and the most commonly received treatments included anthracycline‐ and/or taxane‐based chemotherapy and radiation therapy (see Table 1).

### 3.2 Peak plantar‐flexion exercise and recovery kinetics

**FIGURE 1:**
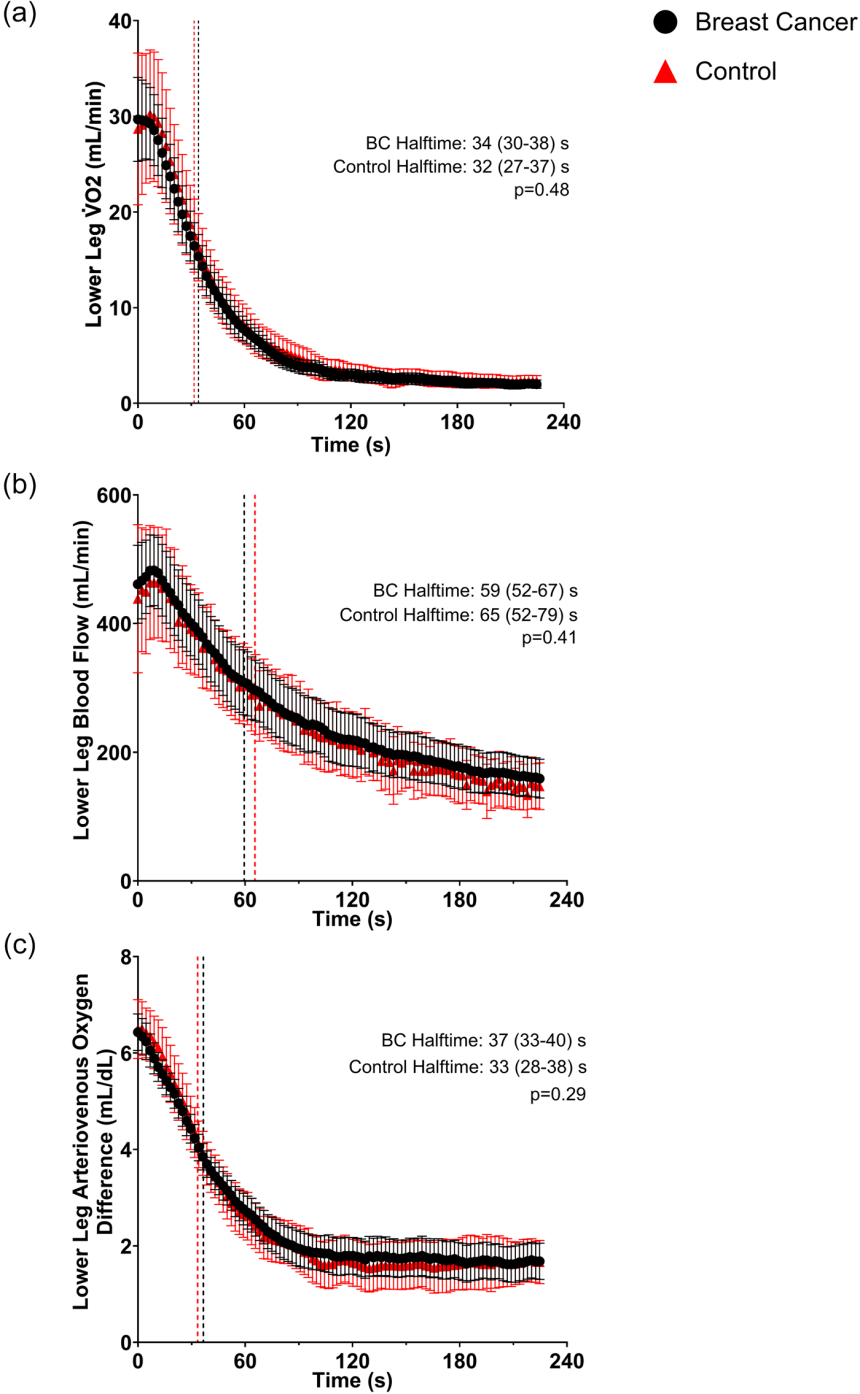
Lower‐leg oxygen uptake (${\dot{V}}_{O_{2}}$), blood flow and arterial venous O_2_ content difference following peak plantar‐flexion exercise in older long‐term breast cancer (BC) survivors and control subjects. Data are group means and 95% confidence intervals.

As shown in Table 2, there were no significant differences between groups for peak plantar‐flexion power output, lower‐leg ${\dot{V}}_{O_{2}}$ and blood flow (absolute values or indexed to calf muscle mass), venous O_2_ saturation or arteriovenous O_2_ difference. The half‐times of recovery for these outcomes were also not significantly different between BCS and CON groups (Figure 1).

**TABLE 2:** Peak plantar‐flexion lower‐leg oxygen uptake, blood flow and arteriovenous O_2_ difference.

Peak plantar‐flexion exercise	BCS	CON	*P*‐value	*d*
Power output, W	12 ± 3	12 ± 2	0.68	0.02
Exercise time, min	5 ± 2	5 ± 1	0.99	–
Lower‐leg ${\dot{V}}_{O_{2}}$, mL/min	31.7 ± 11.7	32.6 ± 13.6	0.81	0.07
Lower‐leg ${\dot{V}}_{O_{2}}$, mL/kg/min^a^	39.7 ± 15.1	39.6 ± 13.4	0.99	0.01
Lower‐leg blood flow, mL/min	507 ± 157	501 ± 171	0.90	0.04
Lower‐leg blood flow, mL/kg/min^a^	645 ± 224	671 ± 295	0.73	0.11
Venous O_2_ saturation, %	61.3 ± 5.7	58.9 ± 6.1	0.17	0.42
Arteriovenous O_2_ difference, mL/dL	6.1 ± 1.1	6.5 ± 0.9	0.21	0.38

*Note*: Abbreviations: BCS, breast cancer survivor; CON, control subject; ${\dot{V}}_{O_{2}}$, oxygen uptake.

^a^ Indexed to calf muscle mass.

### 3.3 Submaximal plantar‐flexion exercise recovery kinetics

**FIGURE 2:**
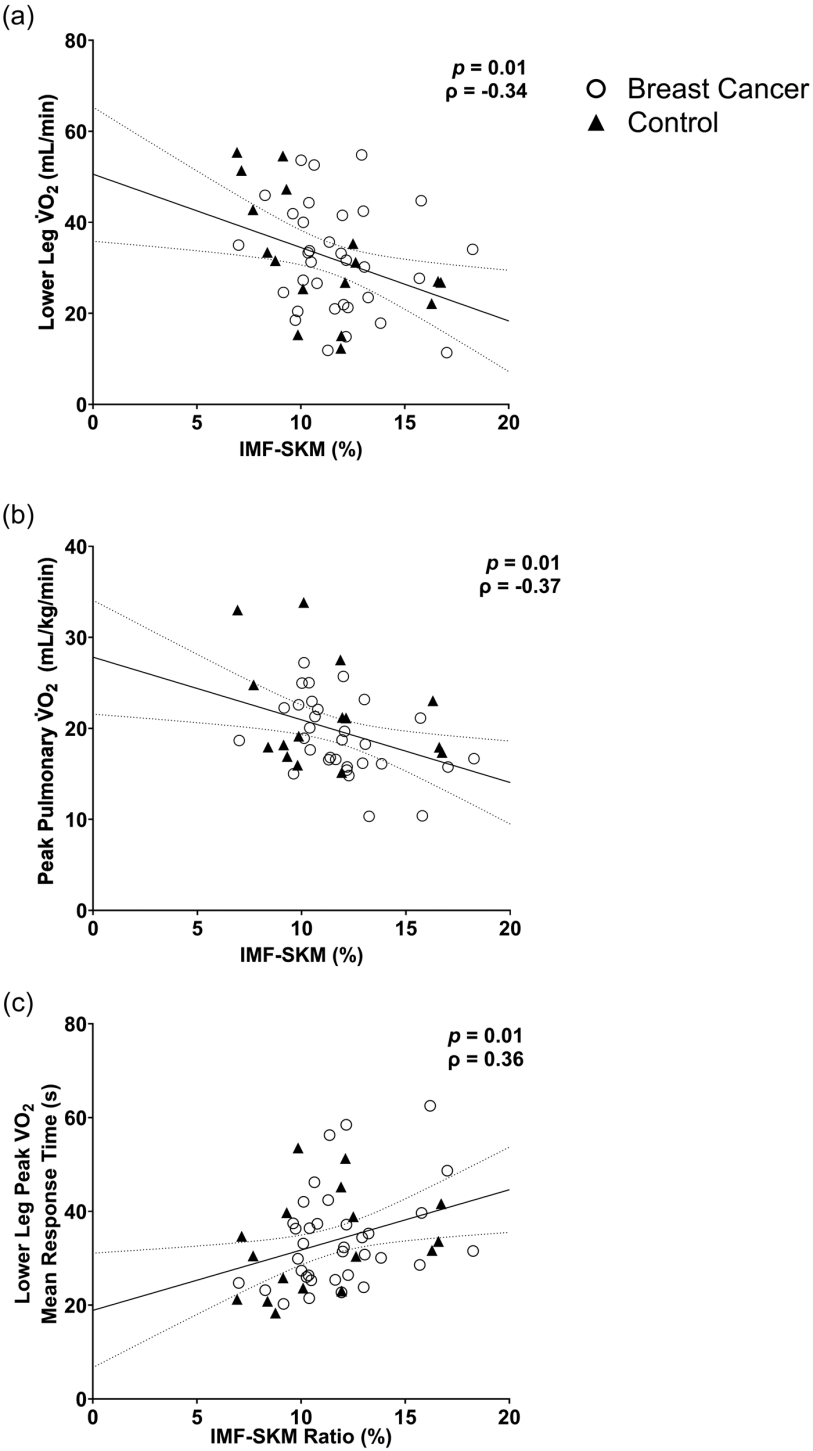
Relationship between myosteatosis and peak pulmonary oxygen uptake (${\dot{V}}_{O_{2}}$) (cycle ergometry), peak lower‐leg ${\dot{V}}_{O_{2}}$ (plantar‐flexion ergometry) and peak lower‐leg ${\dot{V}}_{O_{2}}$ off kinetics in older long‐term breast cancer survivors and control subjects. Abbreviation: IMF/SM, intermuscular fat to skeletal muscle ratio.

Submaximal plantar‐flexion power output, lower‐leg ${\dot{V}}_{O_{2}}$ blood flow and arteriovenous O_2_ difference were not different between groups (Table 3). Furthermore, there were no significant differences in the postexercise half‐time following submaximal plantar‐flexion exercise for lower‐leg ${\dot{V}}_{O_{2}}$ [30 (27–33) vs. 31 (28–34) s, *P* = 0.54], blood flow [44 (35–52) vs. 48 (38–61) s, *P* = 0.29], arteriovenous O_2_ difference [46 (38–55) vs. 39 (28–50) s, *P* = 0.28] or venous O_2_ saturation [46 (37–54) vs. 39 (8–48) s, *P* = 0.09] recovery between BCS and CON, respectively (Figure 2).

**TABLE 3:** Submaximal plantar‐flexion exercise and postexercise recovery half‐time for lower‐leg oxygen uptake, blood flow and arteriovenous O_2_ difference.

Submaximal plantar‐flexion exercise	BCS	CON	*P*‐value	*d*
Power output, W	7 ± 2	7 ± 1	0.15	–
Lower‐leg ${\dot{V}}_{O_{2}}$, mL/min	23.8 ± 7.3	25.0 ± 6.7	0.57	0.17
Lower‐leg ${\dot{V}}_{O_{2}}$, mL/kg/min^a^	30.3 ± 10.2	33.1 ± 13	0.41	0.14
Lower‐leg blood flow, mL/min	393 ± 123	399 ± 116	0.85	0.01
Lower‐leg blood flow, mL/kg/min^a^	503 ± 189	509 ± 136	0.91	0.03
Lower‐leg venous O_2_ saturation, %	61.6 ± 5.6	60.7 ± 4.7	0.58	0.17
Lower‐leg arteriovenous O_2_ difference, mL/dL	6.2 ± 1.0	6.2 ± 0.9	0.88	0.05
Postexercise recovery half‐time				
Lower‐leg ${\dot{V}}_{O_{2}}$, s	30 (27–33)	31 (28–34)	0.54	0.19
Lower‐leg blood flow, s	44 (35–52)	48 (38–61)	0.29	0.24
Lower‐leg venous saturation, s	46 (37–54)	39 (28–48)	0.09	0.33
Lower‐leg arteriovenous O_2_ difference, s	46 (38–55)	39 (28–50)	0.28	0.34

*Note*: Data are the mean ± SD or 95% confidence interval. Abbreviations: BCS, breast cancer survivor; CON, control subject; ${\dot{V}}_{O_{2}}$, oxygen uptake.

^a^ Indexed to calf muscle mass.

### 3.4 Associations between myosteatosis and pulmonary peak ${\dot{V}}_{O_{2}}$ or lower‐leg peak ${\dot{V}}_{O_{2}}$

A significant negative relationship was found between calf myosteatosis and several exercise performance outcomes. Specifically, a higher intermuscular fat to skeletal muscle (IMF/SM) ratio was associated with a lower peak pulmonary ${\dot{V}}_{O_{2}}$ (cycle exercise, ρ = −0.37, *P* = 0.01), lower peak plantar‐flexion exercise power output (ρ = −0.30, *P* = 0.03) and leg ${\dot{V}}_{O_{2}}$ (ρ = −0.34, *P* = 0.01). Also, higher calf myosteatosis was associated with slowed peak plantar‐flexion lower‐leg ${\dot{V}}_{O_{2}}$ (ρ = 0.36, *P* = 0.01) and submaximal plantar‐flexion arteriovenous O_2_ difference recovery kinetics (ρ = 0.30, *P* = 0.04).

## 4 DISCUSSION

This study provides the first comprehensive evaluation of lower‐leg ${\dot{V}}_{O_{2}}$ and recovery kinetics during small muscle mass exercise in older, long‐term BCS. The primary new findings are twofold. First, contrary to our primary hypothesis, we observed no significant differences in lower‐leg ${\dot{V}}_{O_{2}}$, blood flow or arteriovenous O_2_ difference during peak or submaximal plantar‐flexion exercise between BCS and CON subjects. Likewise, the recovery kinetics for these measures did not differ between the groups. Second, our secondary hypothesis was supported, because we found significant associations between calf myosteatosis and various measures of exercise performance outcomes. Specifically, a higher calf muscle IMF/SM ratio was linked to lower peak pulmonary ${\dot{V}}_{O_{2}}$ (cycle exercise) and reduced lower‐leg ${\dot{V}}_{O_{2}}$ during plantar‐flexion exercise, and to slower recovery kinetics for lower‐leg ${\dot{V}}_{O_{2}}$ and O_2_ extraction following peak and submaximal plantar‐flexion exercise in both groups. These findings offer new insights into the potential mechanisms contributing to exercise intolerance in older, long‐term BCS.

### 4.1 Preserved muscle function in older long‐term breast cancer survivors

The finding of preserved muscle blood flow and oxidative capacity (${\dot{V}}_{O_{2}}$) in our older BCS cohort is particularly noteworthy given the extensive literature documenting reduced exercise tolerance in this population. Previous studies have consistently reported reduced peak pulmonary ${\dot{V}}_{O_{2}}$ during whole‐body exercise in women with breast cancer during treatment or in the short‐term period (≤5 years) after completing cancer therapy (Beaudry et al., 2020; Haykowsky et al., 2022; Jones et al., 2012, 2011; Kirkham et al., 2019). In contrast, peak pulmonary ${\dot{V}}_{O_{2}}$ was not different between our BCS cohort and age‐ and sex‐matched healthy control subjects. The disparity between our findings and previous studies might be attributed to our participants being more physically active than the general population of BCS previously studied during or after treatment.

Despite comparable cardiorespiratory fitness levels, peak pulmonary ${\dot{V}}_{O_{2}}$ in both BCS and CON (19.3 ± 4.6 and 21.5 ± 5.9 mL/kg/min, respectively) is only marginally above the ${\dot{V}}_{O_{2}}$ required for full and independent living (equal to 18 mL/kg/min) (Paterson et al., 1999). This finding suggests that many activities of daily living might require near‐maximal effort, highlighting the clinical significance of improving cardiorespiratory fitness in this population even when values are comparable to healthy control subjects.

To date, the prevailing explanation for impaired peak pulmonary ${\dot{V}}_{O_{2}}$ in BCS has focused on treatment‐related left ventricular dysfunction and concomitant reduction in peak and reserve (peak exercise minus rest) cardiac output (Foulkes, Haykowsky et al., 2024; Haykowsky et al., 2022). To isolate the contribution of peripheral mechanisms from central cardiovascular limitations, we used a small muscle mass (plantar‐flexion) exercise protocol that minimized cardiac demands while focusing specifically on the intrinsic capacity of skeletal muscle to deliver and use O_2_ during peak and submaximal plantar‐flexion exercise and during postexercise recovery (Jendzjowsky et al., 2007). Our findings demonstrate that older long‐term BCS maintain normal muscle blood flow and O_2_ extraction across all plantar‐flexion exercise intensities and during recovery. These results extend our prior observations showing preserved skeletal muscle blood flow during small muscle mass exercise (unilateral knee extension or plantar flexion) in middle‐aged and older BCS (Beaudry et al., 2022, 2020). Collectively, these findings might help to explain why some BCS can maintain normal exercise capacity, even when central cardiovascular impairments are present, probably owing to the preservation of favourable peripheral skeletal muscle mechanisms (Foulkes, Haykowsky et al., 2024).

### 4.2 Factors contributing to preserved skeletal muscle function

Several factors might explain the preserved skeletal muscle function observed in our study population. First, our participants were, on average, 14 years post‐treatment, which is a substantially longer follow‐up period than previous investigations (Foulkes, Haykowsky et al., 2024). This extended time frame might have allowed for skeletal muscle recovery and adaptation through repeated use during activities of daily living. It is plausible that acute treatment‐related impairments in skeletal muscle function and morphology (including decreased oxidative fibres and enzymes coupled with capillary rarefaction) (Mijwel et al., 2018) are most pronounced during and immediately following treatment, then gradually resolve over time through compensatory mechanisms.

Second, the slower postexercise venous O_2_ saturation kinetics following submaximal exercise in BCS warrants further discussion. Given that O_2_ extraction is directly proportional to diffusive O_2_ conductance (transport of O_2_ from microvasculature to muscle mitochondria) and inversely related to convective O_2_ delivery (Foulkes, Wagner et al., 2024; Haykowsky et al., 2022), our present and prior findings that muscle blood flow and mitochondrial oxidative capacity are preserved in BCS suggest that impaired microvascular function might be an important contributor to the delayed venous O_2_ saturation kinetics (Beaudry et al., 2023, 2020). Thus, the tendency for slower O_2_ extraction recovery might indicate subtle impairments in microvascular function that were not captured by our other measures. Importantly, these outcomes were not measured directly in the present study but represent a key area for future study.

Third, owing to the small muscle mass involved during plantar flexion and the comparable peak blood flow observed between groups (BCS, 0.65 L/min/kg vs. CON, 0.67 L/min/kg in the calf muscle), any differences in this outcome might become apparent only during exercises that engage a larger muscle mass, such as cycling. Indeed, based on the BCS indexed calf leg blood flow coupled with our prior measurement of peak cycle exercise cardiac output of 9.5 L/min in a different small sample of older BCS (*n* = 9, mean age 67 years; time post‐treatment, 10 years; Beaudry et al., 2022), we might observe convective O_2_ delivery impairment only when ∼15 kg of muscle mass is engaged in exercise (e.g. 9.5 L/min ÷ 0.65 L/min/kg = 15 kg of muscle). This suggests that the functional reserve of the peripheral O_2_ delivery system might be sufficient to maintain performance during activities involving smaller muscle groups but might become limiting during whole‐body exercise that engages a muscle mass ≥15 kg, such as walking or climbing stairs.

### 4.3 Clinical significance of myosteatosis and exercise intolerance

A main finding of this study is the association between increased myosteatosis and worse function across a wide range of muscle oxygenation outcomes. This observation extends our previous work and reinforces the physiological significance of myosteatosis as a key contributor to exercise intolerance in older women, both with and without a history of breast cancer (Beaudry et al., 2020; Haykowsky et al., 2014, 2018).

In the present study, we demonstrated that higher myosteatosis was significantly correlated with decreased peak pulmonary ${\dot{V}}_{O_{2}}$ during cycle exercise. We also observed that greater myosteatosis was associated with reduced lower‐leg ${\dot{V}}_{O_{2}}$ and prolonged muscle oxygenation recovery kinetics following plantar‐flexion exercise. These relationships were evident in both the BCS and CON groups, suggesting that myosteatosis represents a fundamental limitation to exercise capacity in older women, independent of a breast cancer diagnosis. Importantly, the relationships between IMF/SM ratio and lower‐leg ${\dot{V}}_{O_{2}}$ (*r* = −0.33, *P* = 0.04) and recovery kinetics (*r* = 0.32, *P* = 0.04) remained after controlling for age, weekly physical activity or group. However, it should be noted that only ∼12% of the variance in ${\dot{V}}_{O_{2}}$ was explained by the increased IMF/SM ratio. The remainder might be attributable, in part, to alterations in skeletal muscle metabolism that might be uncovered using ^31^P spectroscopy; however, this was not measured in the present study.

Our findings herein are consistent with and extend our previous research. Specifically, we previously reported in a cohort of 16 middle‐aged BCS (mean age, 56 years; 3 months post‐treatment) that thigh and leg myosteatosis was inversely related to peak pulmonary ${\dot{V}}_{O_{2}}$ during cycle exercise and to lower‐leg ${\dot{V}}_{O_{2}}$ and O_2_ extraction during submaximal plantar‐flexion exercise (Beaudry et al., 2020). Likewise, in older heart failure patients with preserved left ventricular ejection fraction (a condition for which older BCS are at elevated risk; Reding et al., 2022), we found that an increased thigh myosteatosis was associated with reduced peak pulmonary ${\dot{V}}_{O_{2}}$ and aerobic endurance (Haykowsky et al., 2005, 2018). The present study builds upon these observations by demonstrating a clear link between myosteatosis and impaired muscle oxygenation and recovery kinetics, providing a more comprehensive picture of the multifaceted mechanisms of exercise intolerance in older, long‐term BCS.

The mechanistic basis for these associations is likely to be multifactorial, involving several pathophysiological processes. Obesity‐ and myosteatosis‐mediated inflammation contribute to endothelial dysfunction, capillary rarefaction and mitochondrial dysfunction (Kitzman & Shah, 2016). These abnormalities collectively compromise both convective and diffusive O_2_ transport to the skeletal muscle, in addition to the capacity of the muscle for oxidative metabolism (Beaudry et al., 2020; Haykowsky et al., 2022). Our finding that myosteatosis was linked specifically to impaired fractional O_2_ extraction and prolonged recovery kinetics suggests that the infiltration of fat into the muscle might directly interfere with the efficient utilization of delivered O_2_. This is particularly evident during the postexercise recovery transition, when the muscle must restore metabolic homeostasis rapidly.

From a functional perspective, the relationship between myosteatosis and prolonged recovery kinetics is particularly significant. The speed of recovery reflects the ability of the muscle to restore metabolic balance efficiently after physical exertion, a process that is essential for performing repeated activities of daily living (Poole & Jones, 2012). Slower recovery from exercise, as observed in individuals with greater myosteatosis, indicates inefficient metabolic transitions, which result greater fatigue during daily activities and necessitate longer rest periods between tasks. Ultimately, this can lead to a decline in overall functional capacity and a diminished quality of life.

### 4.4 Study limitations and future directions

Several limitations of this study should be acknowledged. First, the cross‐sectional design precludes the establishment of causal relationships between myosteatosis and exercise tolerance. Longitudinal studies are needed to determine whether changes in muscle composition over time are associated with corresponding changes in whole‐body and muscle ${\dot{V}}_{O_{2}}$ and postexercise recovery. Second, our breast cancer participants were long‐term survivors who were relatively healthy and physically active, which might limit the generalizability of our findings to the broader population of BCS, particularly those with more recent diagnoses or significant comorbidities. Third, the role of exercise interventions, such as resistance training alone or in combination with aerobic training, to improve cardiorespiratory fitness and myosteatosis in older women across the breast cancer survivorship continuum remain under‐studied (Mijwel et al., 2018). Therefore, future research should focus on the role of exercise and/or diet to improve these outcomes in this population. Fourth, it is possible that admixture of blood from non‐working muscles might have resulted in a higher venous saturation; however, this is unlikely, because venous O_2_ saturation was measured at the region that primarily drains the posterior compartment of the lower leg (gastrocnemius and soleus muscles). We also used dominant and non‐dominant limbs for maximal and submaximal exercise, respectively. It is possible that interlimb (dominant) differences might have affected the prescribed exercise intensity and resulting kinetics. However, all participants performed this test in the same standardized manner, hence any influences would probably have been consistent across participants and study groups. Finally, mechanistic studies examining the cellular and molecular basis of myosteatosis‐related functional impairments could identify new therapeutic targets for exercise and pharmacological interventions.

## 5 CONCLUSION

This study provides new insights into the peripheral mechanisms of exercise intolerance in long‐term BCS. Our findings demonstrate that calf skeletal muscle blood flow and oxidative capacity are remarkably well preserved in older, long‐term survivors. However, we find that myosteatosis is an important determinant of whole‐body and muscle ${\dot{V}}_{O_{2}}$ and muscle oxygenation recovery kinetics, regardless of cancer history. These findings suggest that interventions aimed at enhancing skeletal muscle quality might be particularly effective for improving submaximal and maximal exercise tolerance in older BCS.

## AUTHOR CONTRIBUTIONS

Mark J. Haykowsky, Edith Pituskin and Richard B. Thompson contributed to the conception and design of the experiments. All authors contributed to the data collection, analyses and/or interpretation. Nathan R. Weeldreyer drafted the manuscript and performed the statistical analyses. All authors edited and revised the manuscript. The final version of the manuscript was approved by all authors, who agree to be accountable for all aspects of the work in ensuring that questions related to the accuracy or integrity of any part of the work are appropriately investigated and resolved. All persons designated as authors qualify for authorship, and all those who qualify for authorship are listed.

## CONFLICT OF INTEREST

None declared.

## Data Availability

Data will be made available upon reasonable request.
